# Hyperspectral Imagery Super-Resolution by Adaptive POCS and Blur Metric

**DOI:** 10.3390/s17010082

**Published:** 2017-01-03

**Authors:** Shaoxing Hu, Shuyu Zhang, Aiwu Zhang, Shatuo Chai

**Affiliations:** 1 School of Mechanical Engineering and Automation, Beihang University, Beijing 100191, China; shuyuzhang@buaa.edu.cn; 2 Ministry of Education Key Laboratory of 3D Information Acquisition and Application, Capital Normal University, Beijing 100048, China; zhang_aiwu@126.com; 3 Qinghai Academy of Animal Science and Veterinary Medicine, Qinghai 810016, China; chaishatuo@163.com

**Keywords:** image blur metric, Gabor wavelet transform, weighted POCS, hyperspectral imagery, super-resolution

## Abstract

The spatial resolution of a hyperspectral image is often coarse as the limitations on the imaging hardware. A novel super-resolution reconstruction algorithm for hyperspectral imagery (HSI) via adaptive projection onto convex sets and image blur metric (APOCS-BM) is proposed in this paper to solve these problems. Firstly, a no-reference image blur metric assessment method based on Gabor wavelet transform is utilized to obtain the blur metric of the low-resolution (LR) image. Then, the bound used in the APOCS is automatically calculated via LR image blur metric. Finally, the high-resolution (HR) image is reconstructed by the APOCS method. With the contribution of APOCS and image blur metric, the fixed bound problem in POCS is solved, and the image blur information is utilized during the reconstruction of HR image, which effectively enhances the spatial-spectral information and improves the reconstruction accuracy. The experimental results for the PaviaU, PaviaC and Jinyin Tan datasets indicate that the proposed method not only enhances the spatial resolution, but also preserves HSI spectral information well.

## 1. Introduction

Hyperspectral imagery (HSI) containing about 200 spectral bands in the visible and infrared wavelength regions is an efficient way to describe and store visual information [1,2]. HSI also has a wide range of applications such as terrain classification, mineral detection and exploration, environmental studies, pharmaceutical counterfeiting, and military surveillance, etc. [3,4,5,6,7]. Our focus in this paper will be on the remote sensing field, wherein the spectral images are typically gained by airborne or spaceborne sensors. However, due to high sensitivity in the spectral domain when designing the imaging hardware device, the spatial resolution of the hyperspectral image is often coarser [8]. Therefore, the image super-resolution reconstruction (SRR) technique is utilized to improve the spatial resolution of hyperspectral images. A high-resolution (HR) image is gained from a sequence of observed low-resolution (LR) images through the SRR technique.

The SRR technique was first achieved in the frequency domain by Tsai and Huang [9], who proposed a formulation for the reconstruction of an HR image from LR images. SRR methods based on discrete cosine transform (DCT) [10] or wavelet transform were subsequently proposed [11,12]. However, the frequency domain approaches are hard to combine with the information in the spatial domain. The spectral information of the HR image is also usually difficult to reserve via the DCT- or wavelet transform-based methods. Therefore, many spatial domain-based methods have been developed to overcome these weaknesses in the frequency domain. Interpolation [13], iterative back projection (IBP) [14], Bayesian/maximum a posteriori (MAP) [15] and adaptive filtering [16] methods are typical spatial domain reconstruction methods. Projection onto convex sets (POCS) [17,18] and SRR methods are another spatial domain-based method, but these methods suffer from the fixed bound problem, which affects the accuracy of the HR reconstruction image. Another kind of method is compressive sensing (CS)-based image super-resolution approaches. Kim et al. [19] achieved the SRR via example-based learning and Kernel ridge regression. Yang et al. [20] proposed an SRR approach based on sparse signal representation, whereby a sparse representation for each patch of the low-resolution input is utilized, and then the coefficients of this representation are used to generate the high-resolution output. However, the CS-based methods [21,22] suffer from the data problem, and the SRR results are usually based on the training data scale and image type. The SRR accuracy of CS-based methods is usually low and unstable when the training data scale is small. 

In order to overcome these weaknesses, a novel super-resolution reconstruction algorithm for hyperspectral imagery by APOCS and image blur metric (APOCS-BM) is proposed in this paper. A no-reference image blur metric assessment method [23,24] based on Gabor wavelet transform is utilized to automatically obtain the blur metric of each LR image and patches. Then, the bound in the APOCS is automatically calculated via image blur metric, and the fixed bound problem is solved at the same time. With the contribution of APOCS and image blur metric, the image blur information is also utilized during the reconstruction of the HR image, which improves the accuracy of the reconstructed HR image. The experimental results indicate that the proposed method not only enhances the spatial resolution, but also preserves the HSI spectral information well.

The remainder of the paper is organized as follows. In Section 2, the algorithm steps of the POCS-based SRR method is introduced, and the limitations are discussed. The proposed APOCS-BM is presented in Section 3, and the image blur metric assessment method based on Gabor wavelet transform is also presented in that section. Experimental results are provided in Section 4, and Section 5 concludes the paper.

## 2. Projection onto Convex Set-Based Super-Resolution Reconstruction (SRR)

In mathematics, projections onto convex sets (POCS) is a method to find a point in the intersection of two closed convex sets. Stark and Oskoui [17] first proposed the POCS formulation of super-resolution reconstruction, and the method was also extended by [18]. The POCS-based SRR methods usually utilize an alternative iterative approach to incorporating image prior knowledge about the solution into the reconstruction process. Therefore, the restoration and interpolation problem both can be solved during the estimates of registration parameters. 

According to the basic principle of POCS, incorporating a priori knowledge into the solution is an interpretation of restricting the solution to be a member of a closed convex set $C_{i}$, which is defined as a set of vectors satisfying a particular property. The main purpose of the POCS is to find a vector through the recursion:

(1) $$x^{n+1}=P_{m}P_{m-1}\cdots P_{2}P_{1}x^{n}$$

where the x is the single to be proposed, and it would be the pixel value in the application of SRR. $P_{i}$ is the projection operator which projects the x onto a closed convex set $C_{i}(i=1,2,\ldots m)$, n is a iteration number, and $x^{0}$ is an arbitrary starting point. Then, a data consistency constraint set used in the image SRR for each pixel within the LR images $y_{k}[m_{1},m_{2}]$ is defined as:

(2) $$C_{D}^{k}[m_{1},m_{2}]=\{x[n_{1},n_{2}]:\left|r^{\left(x\right)}[m_{1},m_{2}]\right|\leq \delta_{k}[m_{1},m_{2}]\}$$

where $x[n_{1},n_{2}]$ is the reconstructed pixel value in the HR images, $\delta_{k}[m_{1},m_{2}]$ is a bound (threshold value) reflecting the statistical confidence. $r^{\left(x\right)}[m_{1},m_{2}]$ defined in Equation (3) is the residual error between the pixel value $y_{k}[m_{1},m_{2}]$ and the degradation value of $x[n_{1},n_{2}]$ calculated from a degradation model

(3) $$r^{\left(x\right)}[m_{1},m_{2}]=y_{k}[m_{1},m_{2}]-\sum_{n_{1},n_{2}}x[n_{1},n_{2}]W_{k}[m_{1},m_{2};n_{1},n_{2}]$$

where $W_{k}[m_{1},m_{2};n_{1},n_{2}]$ is a matrix to describe the degradation model via blurring, motion and subsampling operation.

The residual error $r^{\left(x\right)}$ reflects the difference between the reconstructed image and the real image, and the two image pixel values are closer when the residual error is smaller. Therefore, the HR image can be reconstructed by calculating the residual value in Equation (3) and the data consistency constraint convex set in Equation (2). The $\delta_{k}$ in Equation (2) partly determines the results of the reconstructed HR image. As the value of $\delta_{k}$ is large, the possibility of the residual error $r^{\left(x\right)}$ belonging to $\left[-\delta_{k},\delta_{k}\right]$ is high. Then, the number of the correctional pixels by Equation (2) in reconstructed HR image is low, which leads to rough reconstruction results. While the value of $\delta_{k}$ is small, the reconstructed HR image generally suffers the noise problem due to the overflow pixel correction by Equation (2). Most of the $\delta_{k}$ used in the existed POCS-based SRR methods is a fixed value, which affects the accuracy of the HR reconstruction image. In order to overcome these problems, a novel APOCS-BM method combining APOCS and blur metric is proposed in this paper, where the $\delta_{k}$ is adaptive and automatically calculated by the image blur metric.

## 3. Hyperspectral Imagery Super-Resolution by Adaptive Projection onto Convex Sets (APOCS) and Blur Metric

### 3.1. Image Blur Metric Based on Gabor Wavelet Transform

Image blur is a major operation in the degradation model ($W_{k}[m_{1},m_{2};n_{1},n_{2}]$ in Equation (3)), which makes a contribution to the HR reconstruction image. In order to achieve APOCS, a novel image blur metric assessment method based on Gabor wavelet transform is presented in this paper. According to the feature of human vision system model (HVS), the edge and contour information in an image is more sensitive than others. The edge and contour information is a kind of high frequency information in the image processing domain, and they can be extracted from Gabor wavelet transform. Therefore, the presented novel image blur metric assessment method is mainly based on the image frequency information and its statistical features.

**Figure 1:**
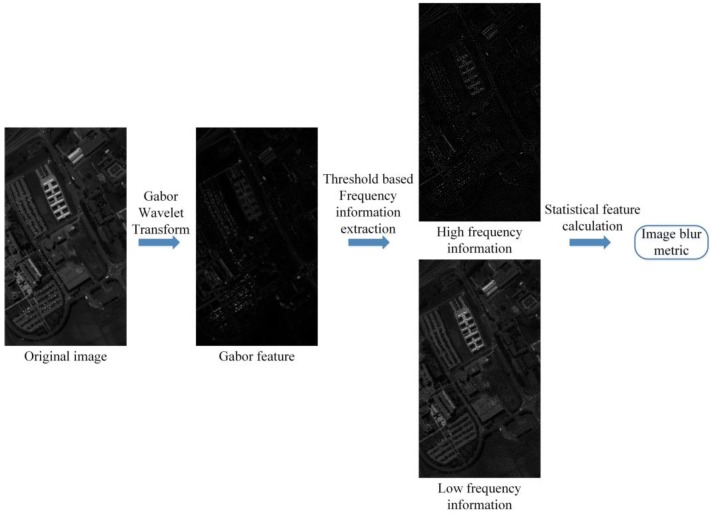
Flowchart of the image blur metric assessment method.

The flowchart of the image blur metric assessment method based on Gabor wavelet transform is shown in Figure 1. The original image ($f(m_{1},m_{2})$) in Figure 1 is from the Pavia university dataset, and the spectral band is 28. The Gabor feature in Figure 1 is extracted by the Gabor wavelet transform, in which the transform kernel function is

(4) $$\begin{array}{l} \psi_{{\vec{k}}_{j}}(\vec{m})=\frac{{\left\|{\vec{k}}_{j}\right\|}^{2}}{\sigma^{2}}\exp(-\frac{{\left\|{\vec{k}}_{j}\right\|}^{2}{\left\|\vec{m}\right\|}^{2}}{2\sigma^{2}})[\exp(i{\vec{k}}_{j}\vec{m})-\exp(-\frac{\sigma^{2}}{2})]\\ {\vec{k}}_{j}=\left(\begin{array}{l} k_{v}\cos\varphi_{u}\\ k_{v}\sin\varphi_{u} \end{array}\right) \end{array}$$

where $k_{v}$, $\varphi_{u}$, and σ are the parameters utilized to gain the frequency and texture feature information, and $\vec{m}$ is the coordinate vector of image pixel. In order to divide the Gabor feature into two classes (high and low frequency), an adaptive threshold-based frequency information extraction method is employed in this paper. Let $GF(m_{1},m_{2})$ be a value of the Gabor feature in Figure 1 with location $\left(m_{1},m_{2}\right)$, then the $GF(m_{1},m_{2})$ mean value $mean(m_{1},m_{2})$ and variance value $\varepsilon (m_{1},m_{2})$ are defined as:

(5) $$mean(m_{1},m_{2})=\frac{1}{p^{2}}\sum_{h=-(p-1)/2}^{(p-1)/2}\sum_{l=-(p-1)/2}^{(p-1)/2}GF(m_{1}+h,m_{2}+l)$$

(6) $$\varepsilon (m_{1},m_{2})=\frac{1}{p^{2}}\sum_{h=-(p-1)/2}^{(p-1)/2}\sum_{l=-(p-1)/2}^{(p-1)/2}\left|GF(m_{1}+h,m_{2}+l)-mean(m_{1},m_{2})\right|$$

where p is the neighborhood size, and it is an odd number. The frequency information in the neighborhood of $GF(m_{1},m_{2})$ is captured by the $mean(m_{1},m_{2})$ and $\varepsilon (m_{1},m_{2})$. Then an adaptive threshold is utilized to achieve the $GF(m_{1},m_{2})$ classification via the captured frequency information. The adaptive threshold $t(m_{1},m_{2})$ is defined as:

(7) $$t(m_{1},m_{2})=mean(m_{1},m_{2})+\varepsilon (m_{1},m_{2})$$

the classification result $C(m_{1},m_{2})$ of the $GF(m_{1},m_{2})$ is defined as:

(8) $$C(m_{1},m_{2})=\left\{ \begin{array}{l} 1,\;if\;GF(m_{1},m_{2})>t(m_{1},m_{2})\\ 0,\;others \end{array}\right.$$

where value 1 in $C(m_{1},m_{2})$ describes the high frequency information, value 0 represents the low frequency information. Let $HF(m_{1},m_{2})=C(m_{1},m_{2})$, and $LF(m_{1},m_{2})=1-HF(m_{1},m_{2})$. Therefore, the high frequency region $HFR(m_{1},m_{2})$ and low frequency region $LFR(m_{1},m_{2})$ in the original image $f(m_{1},m_{2})$ are calculated by

(9) $$\begin{array}{l} HFR(m_{1},m_{2})=HF(m_{1},m_{2})\cdot f(m_{1},m_{2})\\ LFR(m_{1},m_{2})=LF(m_{1},m_{2})\cdot f(m_{1},m_{2})\; \end{array}$$

where (⋅) means the multiplication of two elements in different matrices with the same location.

The separated frequency information is gained from Gabor wavelet transform and the adaptive threshold-based frequency information extraction. In order to calculate the image blur metric automatically, four statistical features extracted from separated frequency information are utilized in our method,: horizontal absolute difference, mean horizontal absolute difference, vertical absolute difference and mean vertical absolute difference. The four statistical features in the high frequency ($HFR_{had}(m_{1},m_{2}),\;HFR_{mhad},\;HFR_{vad}(m_{1},m_{2}),\;HFR_{mvad}$) are defined as:

$$\begin{array}{l} HFR_{had}(m_{1},m_{2})=\left|HFR(m_{1},m_{2}+1)-HFR(m_{1},m_{2}-1)\right|\\ HFR_{mhad}=\frac{1}{MN}\sum_{m_{1}=1}^{M}\sum_{m_{2}=1}^{N}HFR_{had}(m_{1},m_{2}) \end{array}$$

(10) $$HFR_{vad}(m_{1},m_{2})=\left|HFR(m_{1}+1,m_{2})-HFR(m_{1}-1,m_{2})\right|$$

(11) $$HFR_{mvad}=\frac{1}{MN}\sum_{m_{1}=1}^{M}\sum_{m_{2}=1}^{N}HFR_{vad}(m_{1},m_{2})$$

where M,N is the original image size. The four statistical features in the low frequency ($LFR_{had}(m_{1},m_{2}),\;LFR_{mhad},\;LFR_{vad}(m_{1},m_{2}),\;LFR_{mvad}$) are defined as:

(12) $$\begin{array}{l} LFR_{had}(m_{1},m_{2})=\left|LFR(m_{1},m_{2}+1)-LFR(m_{1},m_{2}-1)\right|\\ LFR_{mhad}=\frac{1}{MN}\sum_{m_{1}=1}^{M}\sum_{m_{2}=1}^{N}LFR_{had}(m_{1},m_{2})\\ LFR_{vad}(m_{1},m_{2})=\left|LFR(m_{1}+1,m_{2})-LFR(m_{1}-1,m_{2})\right|\\ LFR_{mvad}=\frac{1}{MN}\sum_{m_{1}=1}^{M}\sum_{m_{2}=1}^{N}LFR_{vad}(m_{1},m_{2})\;. \end{array}$$

The statistical features of separated frequency information are gained by Equations (10) and (11). Combined with these statistical features, an image blur metric assessment ($A_{IBM}$) is utilized to describe the hyperspectral image blur metric in our method:

(13) $$A_{IBM}=\frac{LFR_{mhad}+LFR_{mvad}}{HFR_{mhad}+HFR_{mvad}}$$

**Figure 2:**
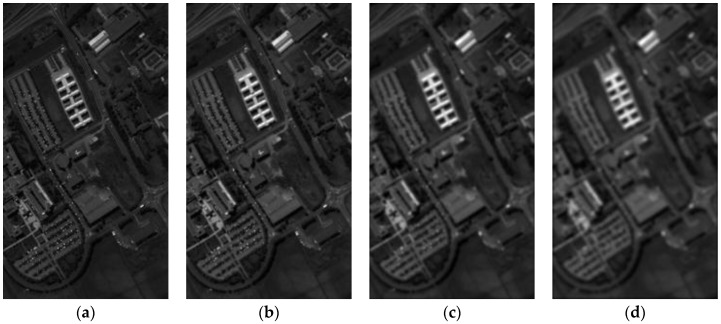
Blur images gained from the Pavia university dataset with a 5 × 5 Gaussian kernel of different standard deviations. (**a**) The standard deviation of Gaussian kernel is 0.1; (**b**) the standard deviation of Gaussian kernel is 0.5; (**c**) the standard deviation of Gaussian kernel is 1; (**d**) the standard deviation of Gaussian kernel is 2. (**a**) $A_{IBM}=1.3588$; (**b**) $A_{IBM}=1.3366$; (**c**) $A_{IBM}=1.2952$; (**d**) $A_{IBM}=1.2473$.

Figure 2 shows $A_{IBM}$ with different blur images, and the spectral band of the blur images is 28. These blur images are gained from the Pavia university dataset with a 5×5 Gaussian kernel of different standard deviations (the standard deviation of Gaussian kernel is 0.1, 0.5, 1 and 2). From the comparison of different blur images, it can be observed that the $A_{IBM}$ is decreasing with the increasing level of image blur, and the blur metric of the image is well described by the proposed method. The Algorithm 1 is the steps of the image blur metric assessment method.

**Algorithm 1.** Steps of the image blur metric assessment method:Step 1: Set the initial value of *p* (Equation (5));Step 2: Compute the Gabor feature *GF*(*m*_1_,*m*_2_) via Equation (4);Step 3: Compute the mean value *m*(*m*_1_,*m*_2_) and variance value *ε*(*m*_1_,*m*_2_) of the Gabor feature with Equations (5) and (6);Step 4: Compute the adaptive threshold *t*(*m*_1_,*m*_2_) (Equation (7)) and achieve the Gabor feature classification (Equation (8));Step 5: Gain the separated frequency information *HFR*(*m*_1_,*m*_2_) and *LFR*(*m*_1_,*m*_2_) via Equation (9);Step 6: Extract the statistical features *HFR_had_*(*m*_1_,*m*_2_), *HFR_mhad_*, *HFR_vad_*(*m*_1_,*m*_2_), *HFR_mvad_* and *LFR_had_*(*m*_1_,*m*_2_), *LFR_mhad_*, *LFR_vad_*(*m*_1_,*m*_2_), *LFR_mvad_* from the separated frequency information (Equations (10) and (11));Step 7: Compute the image blur metric assessment *A_IBM_* via statistical features (Equation (12)).

### 3.2. Proposed APOCS-Blur Metrics (BM) Method

The presented image blur metric assessment $A_{IBM}$ (Equation (12)) in the last subsection is automatically calculated by the statistical features of separated frequency information. With the contribution of $A_{IBM}$, a novel super-resolution reconstruction algorithm for hyperspectral imagery based on adaptive projection onto convex sets and image blur metric is proposed in this paper. The steps of Algorithm 2 are shown below:

**Algorithm 2.** Steps of the proposed APOCS-BM method:Step 1: Set the initial value of *p* (Equation (5)), *α*, *β*, *t*_0_ (Equation (13)) and iteration number *Itn*;Step 2: Gain the initial HR image *H* from the LR image *L*_1_ by linear interpolation, calculate the *A_IBM_*[*m*_1_,*m*_2_] and $A_{IBM}^{LR}$ for each LR image *L*_1_~*L*_4_;Step 3: **For**
*i* = 1,2, …, *Itn*
     **for**
*j* = 1,2, 3, 4       Step 3.1: Calculate the affine motion parameters for LR image *L_j_*;       Step 3.2: Gain the estimation value *H_es_* of *H* via the affine motion parameters and point spread function;       Step 3.3: Calculate the residual *R_j_*(*i*);       Step 3.4: Calculate the adaptive threshold value *δ_k_*[*m*_1_,*m*_2_];       Step 3.5: **If**
*R_j_*(*i*) > *δ_k_*[*m*_1_,*m*_2_] or *R_j_*(*i*) < −*δ_k_*[*m*_1_,*m*_2_]            Step 3.5.1: refresh *H* with the estimation value *H_es_*;           **end If**     **end for**    **end for**Step 4: output the reconstructed HR image *H*.

**Figure 3:**
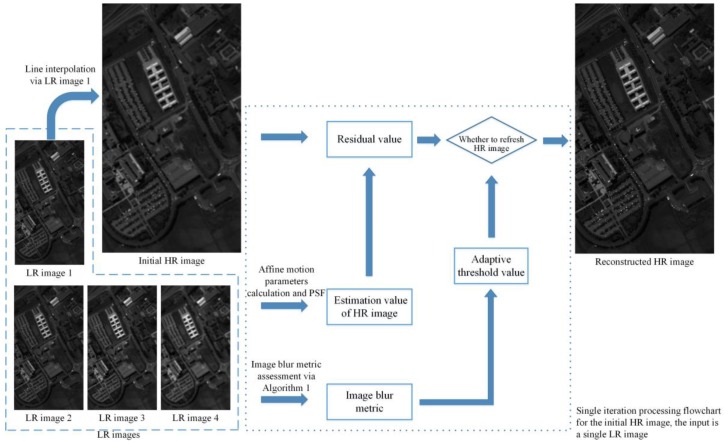
Flowchart of the single iteration processing for the proposed APOCS-BM method.

The input of the proposed APOCS-BM method is four LR images $L_{1}\sim L_{4}$, and an iteration operation (in Step 3) is employed to improve the accuracy of the reconstructed HR image. The initial HR image H is calculated from $L_{1}$ via a linear interpolation operation. In the steps of calculating the HR image estimation value $H_{es}$ (Steps 3.1 and 3.2), affine motion parameters and a point spread function are utilized. Then the residual $R_{j}(i)$ between estimation value $H_{es}$ and the initial HR image H is gained. The adaptive threshold value $\delta_{k}[m_{1},m_{2}]$ (Equation (2)) used in the APOCS-BM method is defined as:

(14) $$\delta_{k}[m_{1},m_{2}]=\alpha \cdot \frac{A_{IBM}[m_{1},m_{2}]}{A_{IBM}^{LR}}\cdot t_{0}+\beta$$

where $A_{IBM}[m_{1},m_{2}]$ is the blur metric assessment of an image patch with center location $\left(m_{1},m_{2}\right)$. The image patch size is 8 × 8 in our method. $A_{IBM}^{LR}$ is the blur metric assessment of the LR image, α is the weight coefficient, β is the correction factor, and $t_{0}$ is a threshold value. Finally, the reconstructed HR image H is refreshed by Step 3.5. In order to describe the algorithm in more detail, Figure 3 presents a flowchart of the single iteration processing for the proposed APOCS-BM method.

The image used in Figure 3 is from the Pavia university dataset with the same spectral band in Figure 2. In Figure 3, the input of the proposed APOCS-BM method is four LR images; the LR image 1 is down-sampled from the original hyperspectral imagery. LR images 2, 3 and 4 are gained from LR image 1 convolving with a 5 × 5 Gaussian kernel of standard deviation 0.1, 0.2 and 0.5, respectively. The initial HR image used in Figure 3 is calculated from LR image 1 via a linear interpolation operation. Then the iteration processing from LR image 1 to LR image 4 is utilized to update the initial HR image. The input of each iteration processing is the output (HR image) of each last iteration, and the iteration processing order is LR images 1, 2, 3 and 4. The reconstructed HR image is refreshed by the iteration processing of four LR images. It can be observed that the spatial information and visual details in the HR image are effectively recovered by the proposed APOCS-BM method. 

In the proposed method, the patch size selection in the calculation of $A_{IBM}$ is mainly determined by the size of LR image. All the LR image sizes used in the experiment are 128 × 128. In comparing with other sizes, we found that the image patch 8 × 8 has the best performance in our method. In some other POCS-based SRR methods, the number and the LR image scale factor of inputs can be increased. However, the time cost is also increased, and the robustness and reliability in these algorithms are hard to be maintained. In order to speed up the algorithm, the initial HR image H is gained from the LR image $L_{1}$ by linear interpolation. If the initial estimation of H is set to 0, the reconstructed result is very close to the original one, and only the iteration number Itn should be larger than usual.

## 4. Experiments and Results

To evaluate the performance of the proposed APOCS-BM method, a series of experiments are performed on the Pavia and Jinyin Tan databases. All our experiments are done using MATLAB R2016b (Mathworks Corporation, MA, USA)on a 3.1 GHz Intel i5-2400 with 16GB RAM. The Pavia database consists of the Pavia University (PaviaU) scene and Pavia Centre (PaviaC) scene, which were captured by ROSIS sensor (German Aerospace Center (DLR), Cologne, Germany) during a flight campaign over Pavia in northern Italy. Part of the channels are removed due to noise; the number of spectral bands is 102 for PaviaC and 103 for PaviaU. The Jinyin Tan dataset is a scene of Jinyin Tan, a grassland located in Qinghai province, western China, which was captured by an airborne sensor named Lantian [25,26]. The number of spectral bands is 103. The image size of all HR used in the experiment is 256 × 256 pixels, which is part of the original dataset, and the image size of LR is 128 × 128 pixels. In order to evaluate the algorithm fairly, average peak signal noise ratio (A-PSNR), average structural similarity (A-SSIM) and spectral angle mapper (SAM) are employed as quality indexes. The A-PSNR and A-SSIM are calculated from the average value of whole spectral bands. The SAM represents the spectral distortion between the original and reconstructed HR image by absolute angles. The value of SAM should be zero when the reconstructed HR image is the same as the original. In the experiment, the proposed method is also compared with the linear interpolation method, DCT-based method [10], Kim [19], POCS [17] and sparse representation-based SR (SR-SR) method [20].

### 4.1. PaviaU and PaviaC Dataset

In the proposed APOCS-BM method, the original input is a single LR image (marked as LR image 1 in Figure 3) with a size of 128×128 pixels. The first step of the proposed method is to obtain another three LR images (marked as LR image 1, LR image 2 and LR image 3 in Figure 3), which are gained from the original input LR image convolving with a 5 × 5 Gaussian kernel of standard deviation 0.1, 0.2 and 0.5, respectively. Then, the four LR images are utilized to reconstruct the HR image. In the experiment, in order to achieve the universal property of the proposed method on different datasets, the patch size (p), iteration number (Itn) and $t_{0}$ are all the same, where p=5, Itn=2 and $t_{0}=1$.

(a) PaviaU dataset results

**Figure 4:**
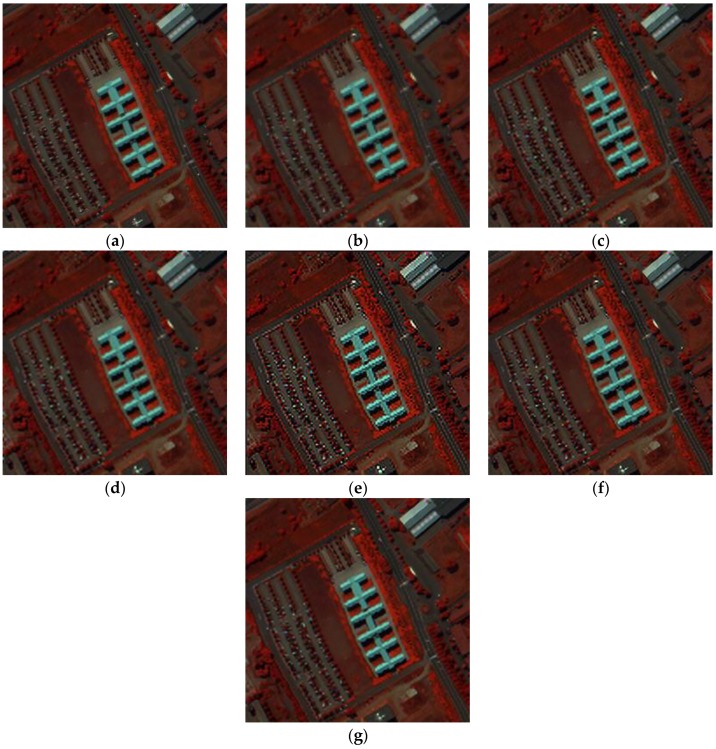
False color image of experimental results for the PaviaU dataset (color composite of R: 80, G: 28, B: 9). (**a**) original HR image; (**b**) linear interpolation results; (**c**) DCT-based method [10] results; (**d**) Kim [19] results; (**e**) POCS [17] results; (**f**) SRSR [20] results; (**g**) proposed APOCS-BM method results.

The other parameters used in the PaviaU dataset are: α=0.8, β=1. Figure 4 shows the visual results of the PaviaU HSI dataset using different SRR methods. For the purpose of visualization by a human observer, the 80th, 28th and 9th spectral bands of the PaviaU dataset are chosen as the R, G and B channels of the color images in Figure 4. It can be observed that the results gained by linear interpolation method or DCT-based method [10] are blurrier than the others. The POSC [17]-based results in Figure 4e are over-sharpened, and the edges and corners are partly changed. From the visual comparison in Figure 4, it can be seen that the proposed APOCS-BM method achieves better spatial-spectral information recovery than the other methods.

**Figure 5:**
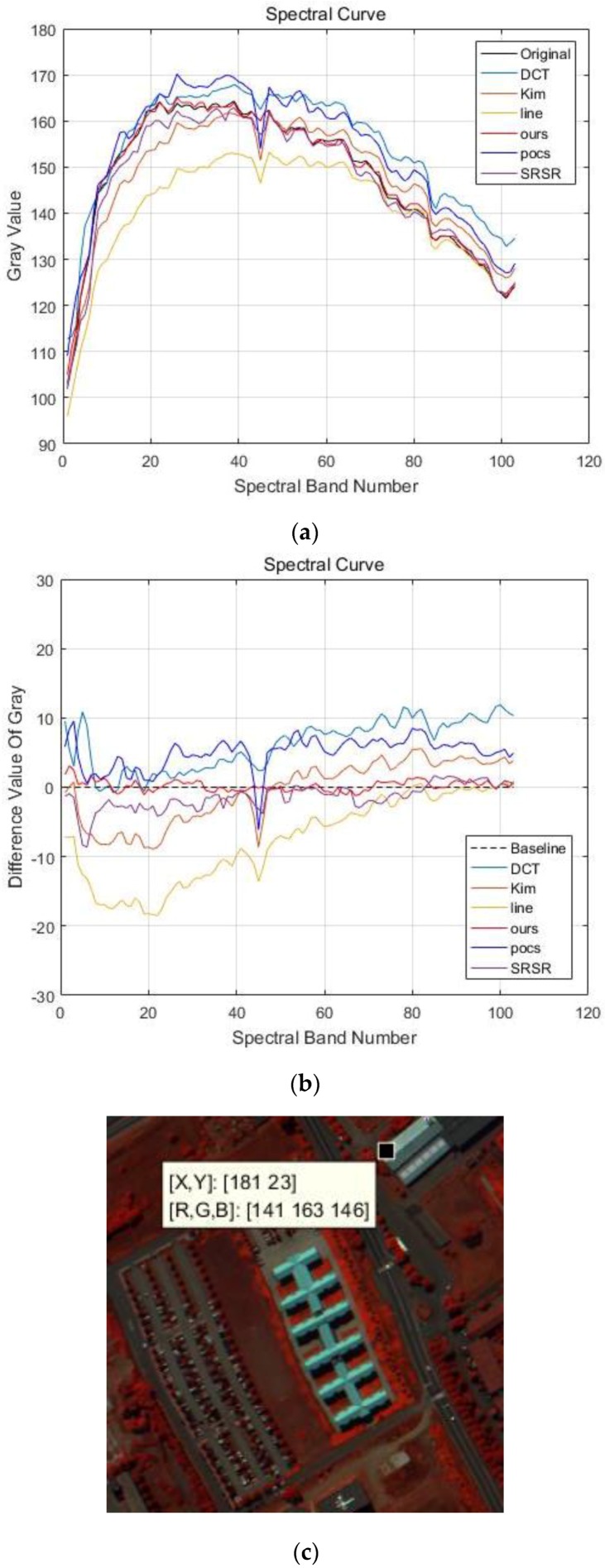
Spectral curves of pixel (181, 23) in the PaviaU dataset. (**a**) Spectral curves of different methods; (**b**) Difference values of spectral curves (the black dotted line is the baseline, the red line represents the results of the proposed methods); (**c**) spectral curve location.

In order to further compare the results with other methods, Figure 5a shows the spectral curves of reconstructed HR images (all spectral images). The horizontal axis is the spectral number, and the vertical axis is the gray value of the spectral image in the same coordinate (the coordinate located at (181,23), shown in Figure 5c). The difference values between the reconstructed spectral curve and the original spectral curve are presented in Figure 5b, the baseline represented by a black dotted line is the original spectral curve. It can be observed that the closer the spectral curves to the baseline, the better the result. From the comparison of different spectral curves in Figure 5, the spectral curve reconstructed by the proposed APOCS-BM method is the best of all reconstructed spectral curves. Table 1 shows the A-PSNR, A-SSIM and SAM of different reconstructed results. We can find that the proposed method has better performance than A-PSNR, A-SSIM and SAM. 

(b) PaviaC dataset results

**Figure 6:**
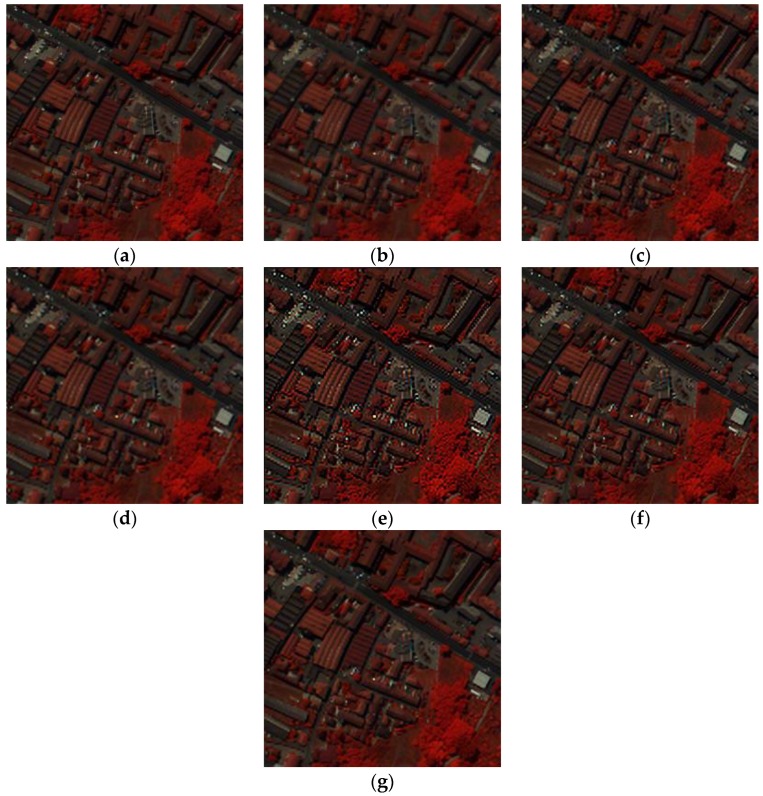
False color image of experimental results for the PaviaC dataset (color composite of R: 80, G: 28, B: 9). (**a**) original HR image; (**b**) linear interpolation results; (**c**) DCT-based method [10] results; (**d**) Kim [19] results; (**e**) POCS [17] results; (**f**) SRSR [20] results; (**g**) proposed APOCS-BM method results.

**Figure 7:**
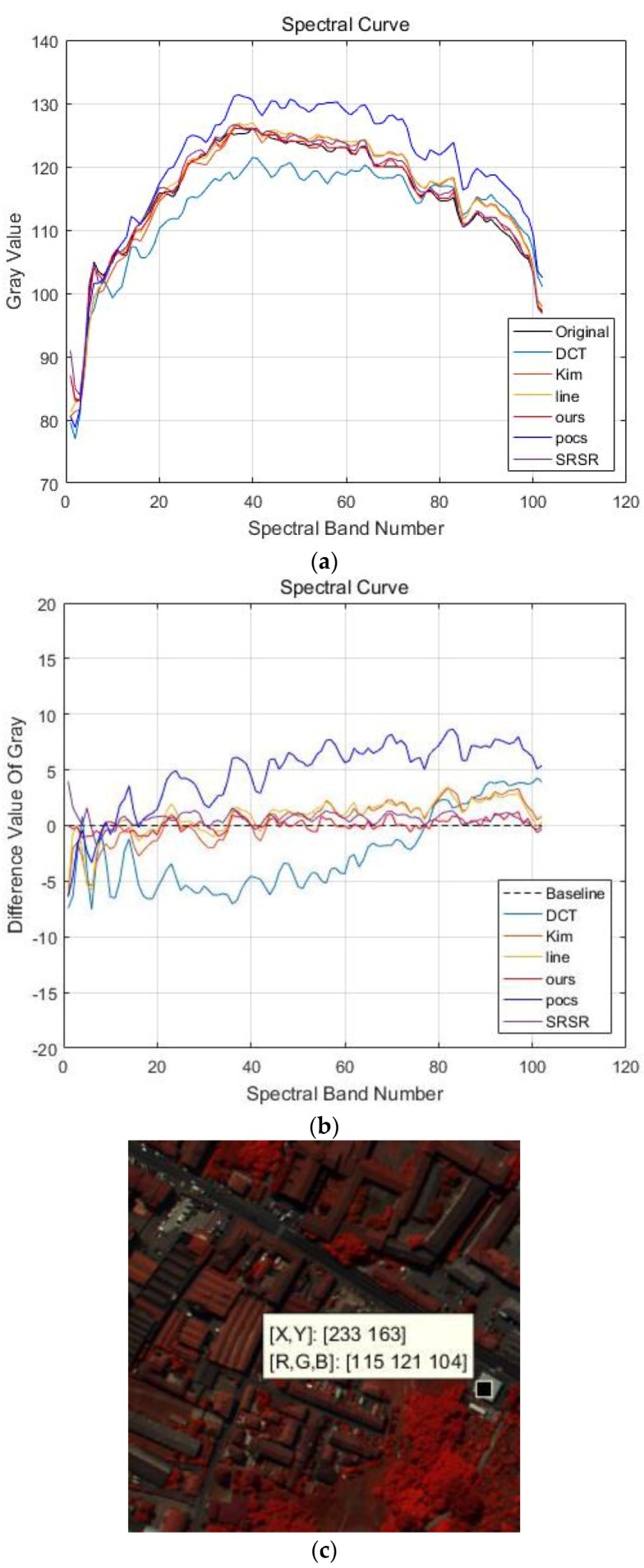
Spectral curves of pixel (233,163) in the PaviaC dataset. (**a**) Spectral curves of different methods; (**b**) Difference values of spectral curves (the black dotted line is the baseline, the red line illustrates the results of the proposed methods); (**c**) spectral curve location.

The parameters used in the PaviaC dataset are: α=1, β=1. The false color image of experimental results for the PaviaC dataset are shown in Figure 6, and the spectral band numbers chosen to be the R, G and B channels are the same as PaviaU. The spectral curves with location (233, 163) are shown in Figure 7. From the comparison of false color image and spectral curves, the reconstructed HR images via the proposed APOCS-BM method contain more spatial-spectral information than the other aforementioned methods. The mean difference value in Figure 7b is smaller than the value in Figure 5b, which is mainly caused by the different material properties. The A-PSNR, A-SSIM and SAM results are shown in Table 2; similar to Table 1, the proposed method performs the best in the different quality indexes.

### 4.2. Jinyin Tan Dataset

**Figure 8:**
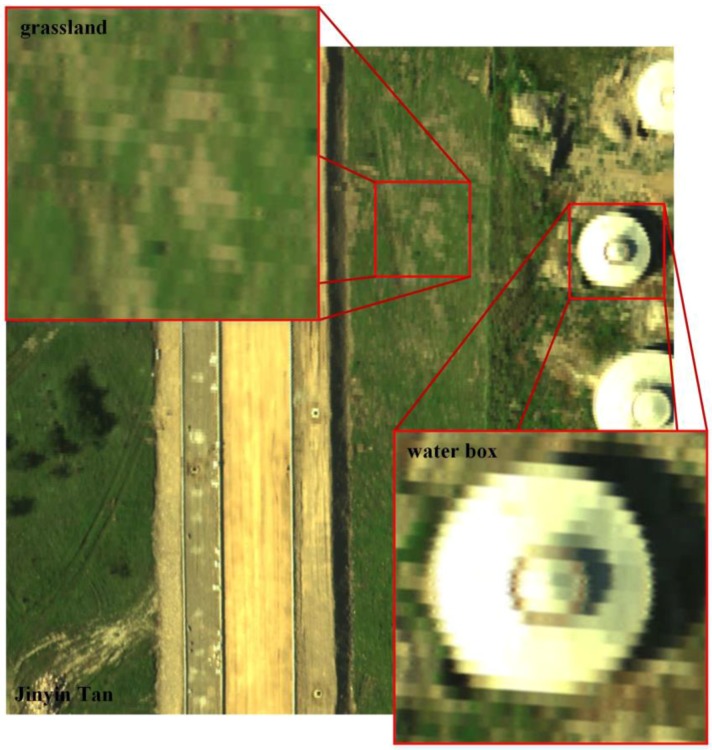
False color image of Jinyin Tan dataset 1 (water box) and Jinyin Tan dataset 2 (grassland) captured from the Jinyin Tan dataset, color composite of R: 48, G: 30, B: 11.

**Figure 9:**
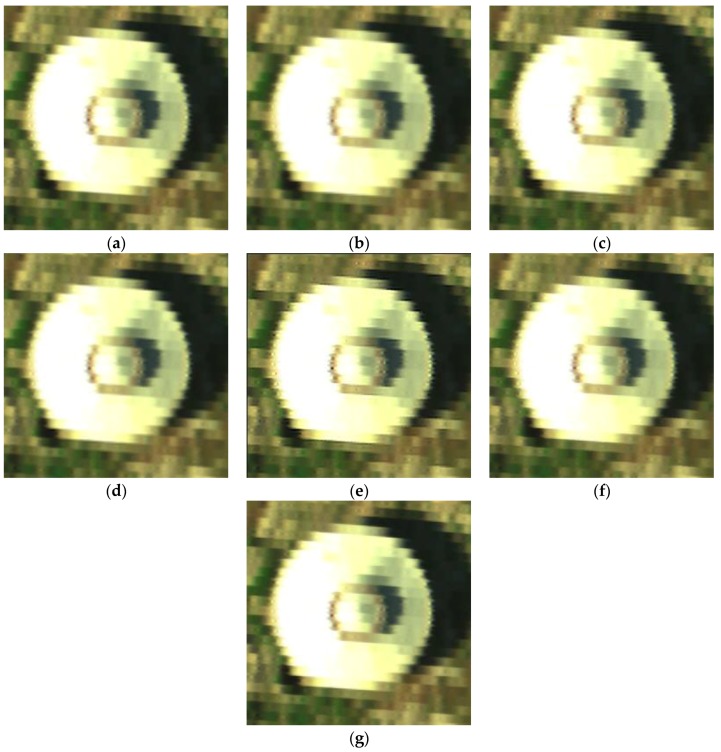
False color image of experimental results for the Jinyin Tan dataset 1 (color composite of R: 48, G: 30, B: 11). (**a**) original HR image; (**b**) linear interpolation results; (**c**) DCT-based method [10] results; (**d**) Kim [19] results; (**e**) POCS [17] results; (**f**) SRSR [20] results; (**g**) proposed APOCS-BM method results.

The Jinyin Tan dataset was captured by the airborne sensor Lantian [21,22]. The Jinyin Tan dataset 1 (the main scene is a water box) and Jinyin Tan dataset 2 (the main scene is grassland) comprise the Jinyin Tan dataset. The details are shown in Figure 8, and the whole image size of the Jinyin Tan is 1681×1681 pixels. The 48th, 30th and 11th spectral bands in the Jinyin Tan dataset 1 are chosen as the R, G and B channels of the false color images in Figure 8 and Figure 9. The parameters used in the two datasets are: α=1.2, β=1, which have the best performance in the experiment. α and β affect the calculation of the adaptive threshold value $\delta_{k}$, which is used in APOCS. When α and β are increased, the adaptive threshold value is increased. In Algorithm 2’s Step 3.5, the amount of the pixel refreshed in H is decreased, but the accuracy of the constructed result (HR image) may be low. When these parameters are decreased, the amount of the pixel refreshed in H is increased, but the noise may be added through this increase. Therefore, α and β are different with different applications.

(a) Jinyin Tan dataset 1 (water box)

**Figure 10:**
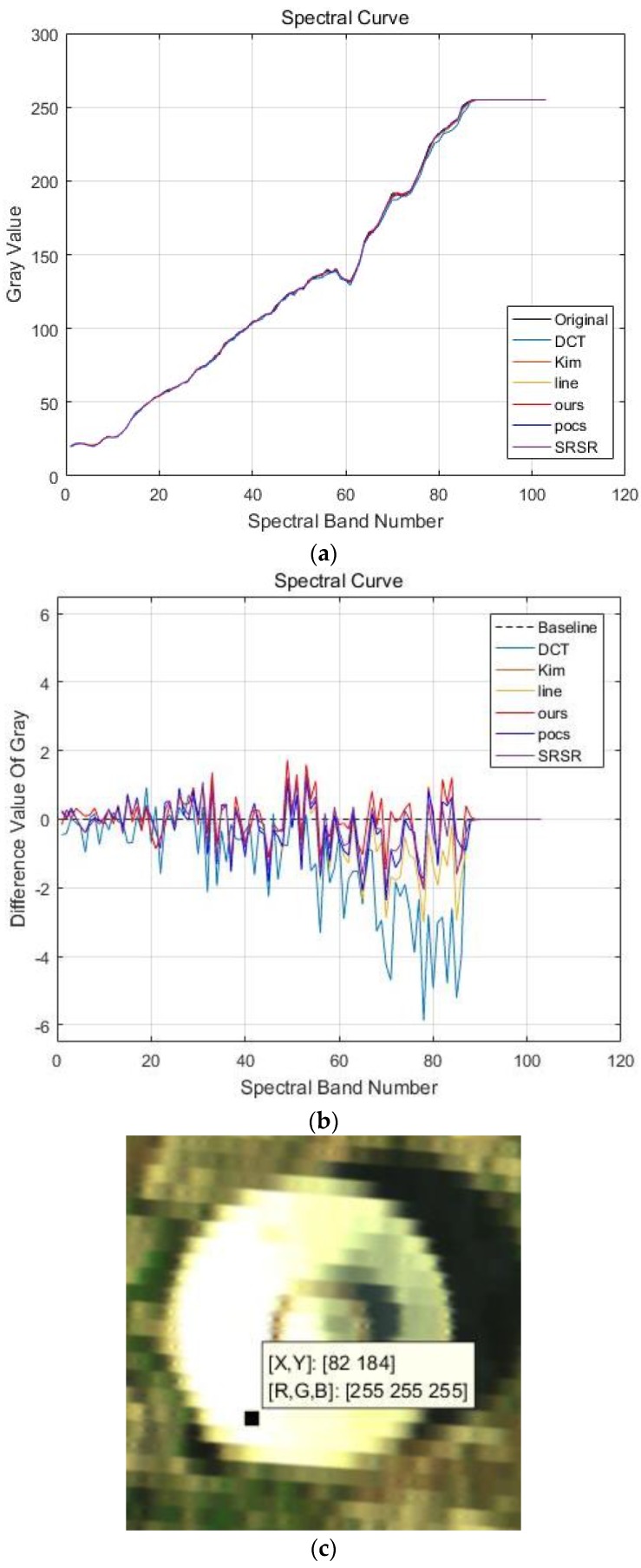
Spectral curves of pixel (82, 184) in the Jinyin Tan dataset 1. (**a**) Spectral curves of different methods; (**b**) Difference values of spectral curves (the black dotted line is the baseline, the red line represents the results of the proposed methods); (**c**) spectral curve location.

The false color images of experimental results for the Jinyin Tan dataset 1 are shown in Figure 9, and the spectral curves with location (82, 184) are shown in Figure 10. The difference value in Figure 10b is much smaller than the values in Figure 5b and Figure 7b, which has a better reconstruction performance in the Jinyin Tan dataset 1. From the visual comparison in Figure 9, we can see that the corners and image texture information of the water box obtained by the proposed method are much better than for the others. It also can be observed that the spatial-spectral information gained via the proposed method is closer to the original signals from Figure 10. Table 3 shows the A-PSNR, A-SSIM and SAM of different experimental results for the Jinyin Tan dataset 1. The A-PSNR in the proposed method is 44.7879, and the constructed HR image is really close to the original HR image. The SAM in the proposed method is 0.0411, which means the spectral distortion between the original and reconstructed HR image is really small. The quality indexes in Table 3 prove that the proposed APOCS-BM method performs better than the others in the Jinyin Tan 1 dataset.

(b) Jinyin Tan dataset 2 (grassland)

**Figure 11:**
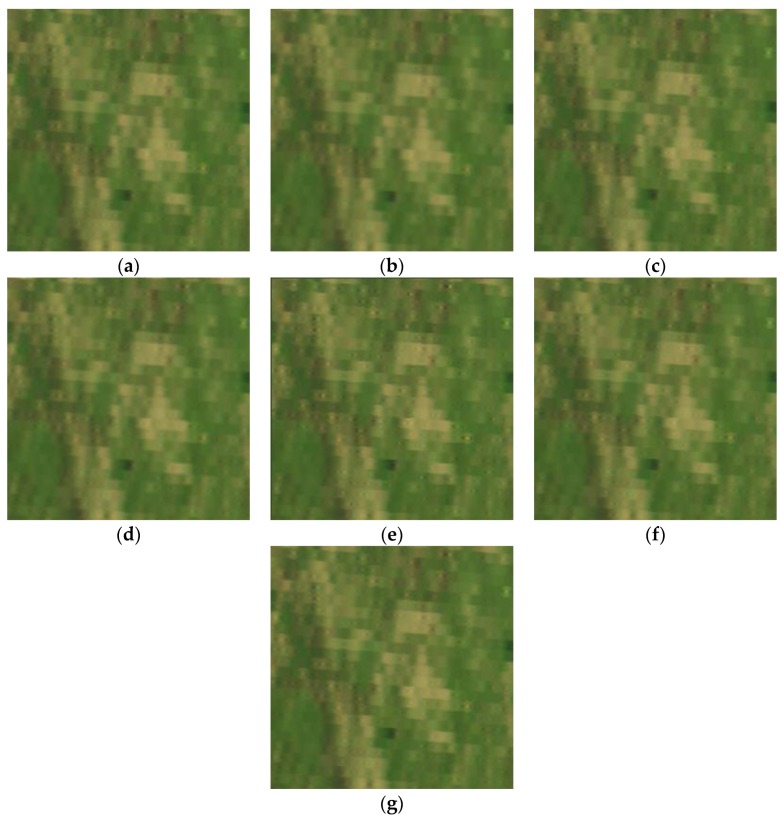
False color image of experimental results for the Jinyin Tan dataset 2 (color composite of R: 48, G: 30, B: 11). (**a**) original HR image; (**b**) linear interpolation results; (**c**) DCT-based method [10] results; (**d**) Kim [19] results; (**e**) POCS [17] results; (**f**) SRSR [20] results; (**g**) proposed APOCS-BM method results.

**Figure 12:**
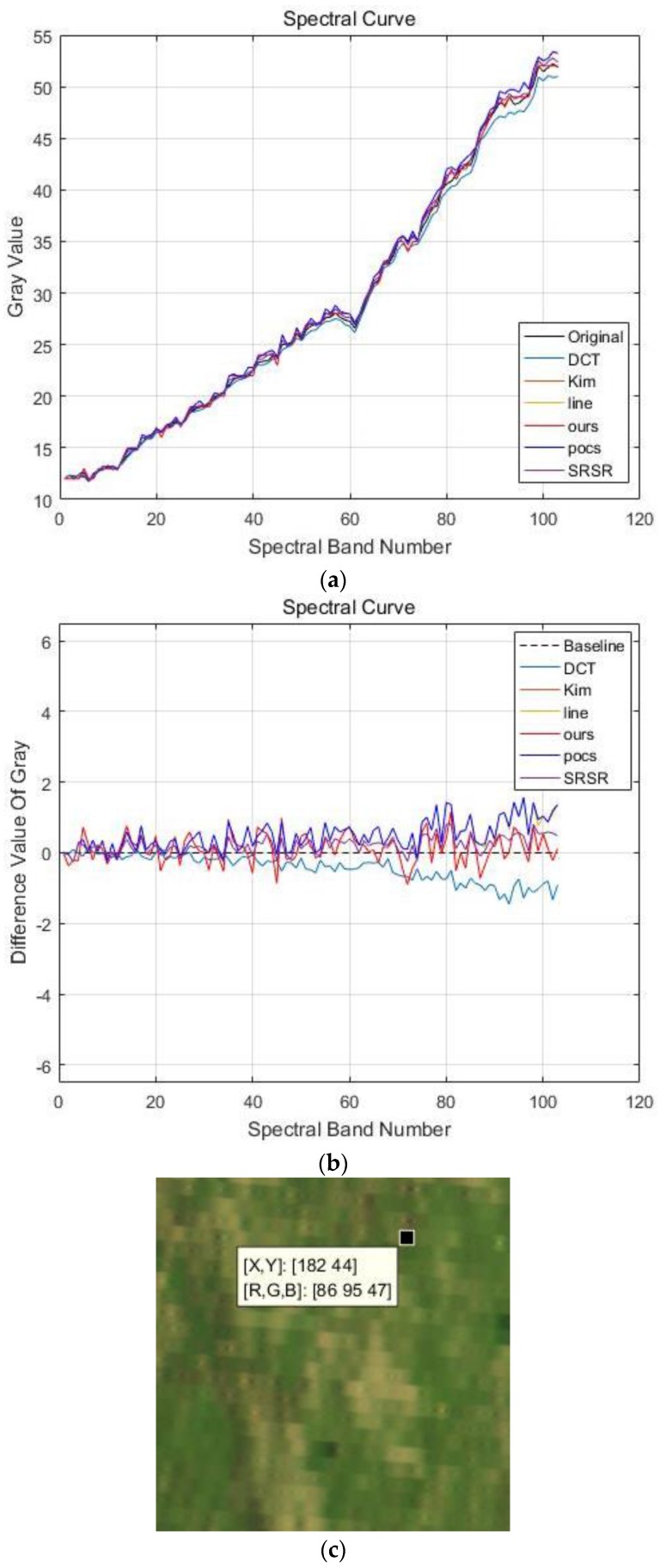
Spectral curves of pixel (182, 44) in the Jinyin Tan dataset 2. (**a**) Spectral curves of different methods; (**b**) Difference values of spectral curves (the black dotted line is the baseline, the red line shows the results of the proposed methods); (**c**) spectral curve location.

Like the Jinyin Tan dataset 1, the false color images of experimental results for the Jinyin Tan dataset 2 are shown in Figure 11, the spectral curves with location (182, 44) are shown in Figure 12, and the quality indexes are shown in Table 4. The 48th, 30th and 11th spectral bands are chosen as the R, G and B channels of the false color images in Figure 11. It can be observed that the difference value in Figure 12b is smaller than 2, and the reconstructed HR images in the Jinyin Tan dataset 2 have the best performance of difference value. From the comparison in the figures and quality indexes, we can see that the reconstructed HR images by the proposed method are much better than by the others, and the spatial-spectral information is well enhanced.

In the experiment, we also compared the execution times of different methods. Table 5 shows the average execution time of different methods for the PaviaU dataset, PaviaC dataset, Jinyin Tan dataset 1 and Jinyin Tan dataset 2. The average execution time is for the reconstruction of a single HR image, not the whole spectral band. It can be observed that the average execution times in line interpolation, DCT-based method [10] and Kim [19] are close, and line interpolation is the fastest. However, the results gained by these methods do not perform well in the comparison of visual or spectral curves. The average execution times in the proposed method and POSC [17] are close, but they are much slower than line interpolation method. The SR-SR method [20] suffers the largest execution time, the main reason being the big dictionary used in the sparse coding and reconstruction. Considering the execution time and reconstruction accuracy of the HR image, the proposed method has the best performance.

## 5. Conclusions

In this paper, a novel super-resolution reconstruction algorithm for hyperspectral imagery via adaptive projection onto convex sets and image blur metric is proposed. In the step of assessing the low resolution (LR) image blur metric, a no-reference image blur metric assessment method based on the Gabor wavelet transform is utilized. Then, the bound (Equation (13)) is automatically calculated via image blur metric. Finally, the high resolution (HR) image is reconstructed by the adaptive projection onto convex sets (APOCS) method. The fixed bound problem in POCS is efficiently solved by the no-reference image blur metric assessment method. With the contribution of APOCS and image blur metric, the image blur information is utilized during the reconstruction of the HR image, which enhances the spatial-spectral information and effectively improves the reconstruction accuracy. The experimental results for the PaviaU, PaviaC and Jinyin Tan datasets indicate that the proposed method not only enhances spatial resolution, but also preserves the hyperspectral imagery (his) spectral information well. Planned future work includes: (i) further improving the spatial resolution and reconstruction accuracy; and (ii) achieving the hyperspectral imagery super-resolution via Convolutional Neural Network (CNN).

## Figures and Tables

**Table 1:** Comparison results of A-PSNR, A-SSIM and SAM on the PaviaU dataset.

Measures	Linear Interpolation	DCT-Based Method [10]	Kim [19]	POCS [17]	SR-SR Method [20]	Proposed Method
A-PSNR	20.8478	24.9727	25.1193	25.7390	26.9570	28.5050
A-SSIM	0.5347	0.6824	0.7108	0.7161	0.8069	0.8435
SAM	0.2148	0.1192	0.1100	0.1002	0.1016	0.0817

**Table 2:** Comparison results of A-PSNR, A-SSIM and SAM for the PaviaC dataset.

Measures	Linear Interpolation	DCT-Based Method [10]	Kim [19]	POCS [17]	SR-SR Method [20]	Proposed Method
A-PSNR	21.4309	25.6022	25.7052	26.3187	27.3867	29.0013
A-SSIM	0.4959	0.6539	0.6761	0.6816	0.7916	0.8292
SAM	0.2633	0.1333	0.1225	0.1093	0.1208	0.0949

**Table 3:** Comparison results of A-PSNR, A-SSIM and SAM on Jinyin Tan dataset 1.

Measures	Linear Interpolation	DCT-Based Method [10]	Kim [19]	POCS [17]	SR-SR Method [20]	Proposed Method
A-PSNR	37.9661	40.0457	40.0424	40.1739	43.8014	44.7879
A-SSIM	0.9608	0.9696	0.9720	0.9727	0.9869	0.9885
SAM	0.0876	0.0621	0.0600	0.0572	0.0588	0.0411

**Table 4:** Comparison results of A-PSNR, A-SSIM and SAM in the Jinyin Tan dataset 2.

Measures	Line Interpolation	DCT-Based Method [10]	Kim [19]	POCS [17]	SR-SR Method [20]	Proposed Method
A-PSNR	47.3629	52.4696	50.7218	50.7808	51.1665	55.5370
A-SSIM	0.9897	0.9905	0.9910	0.9914	0.9947	0.9966
SAM	0.0610	0.0544	0.0474	0.0488	0.0448	0.0405

**Table 5:** Average execution time of different methods for all datasets (single HR image).

Method	Line Interpolation	DCT-Based Method [10]	Kim [19]	POCS [17]	SR-SR Method [20]	Proposed Method
Average time(s)	0.0024	0.0073	0.0597	1.7011	12.2114	1.8414
